# Sequential occurrence of dyspnea at the end of life in palliative care, according to the underlying cancer

**DOI:** 10.1002/cam4.419

**Published:** 2015-01-30

**Authors:** Frédéric Guirimand, Marine Sahut d'izarn, Lucy Laporte, Marie Francillard, Jean-François Richard, Philippe Aegerter

**Affiliations:** 1 Pôle Recherche SPES (“Soins Palliatifs En Société”), Maison Médicale Jeanne GarnierParis, 75015, France; 2 Service de pneumologie et oncologie thoracique, AP-HP, Hôpital Ambroise ParéBoulogne-Billancourt, 92100, France; 3 Université Versailles St-QuentinParis, France; 4 Département de Santé Publique, AP-HP, Hôpital Ambroise ParéBoulogne-Billancourt, 92100, France; 5 UPRES EA 2506, Université Versailles St-QuentinParis, 75016, France

**Keywords:** Cancer, dyspnea, palliative care, palliative care hospital, supportive care

## Abstract

Dyspnea is a symptom that severely affects the quality of life of terminally ill patients. Its frequency differs considerably between studies. We aimed to characterize the frequency of dyspnea in a palliative care hospital (PCH) and to identify factors predisposing to dyspnea, particularly during the very last days of life, as a function of the underlying disease. Episodes of dyspnea were identified by the computerized extraction of prospectively collected data from the reports of care assistants or from medical observations recorded in the medical files for all stays at our PCH during the last 6 years. There were 6455 hospital stays, 88% ending in the death of the patient; 13,282 episodes of dyspnea were recorded during 2608 hospital stays (40%). Dyspnea was more frequently observed in cases of cancer than in other conditions (RR = 1.30; 95% CI: 1.14–1.48). Pulmonary metastasis increased the risk of dyspnea from 37% to 51% (RR = 1.37; 95% CI: 1.29–1.46). Dyspnea frequency varied with the primary cancer site, from 24% (brain cancer) to 60% (esophageal cancer). The data for cancer patients staying for more than 6 days who subsequently died indicated that 8% of patients experienced dyspnea exclusively during the last 4 days of the life, independently of the site of the primary cancer. Dyspnea during the last few days of life requires systematic assessment. Exclusively terminal dyspnea should be distinguished from more precocious dyspnea, as the pathophysiological mechanisms and treatments of these two forms are probably different.

## Introduction

Dyspnea is a complex subjective symptom that impairs the quality of life of terminally ill patients 1–3. It is frequently associated with other symptoms, such as anxiety, insomnia, and asthenia, and is more difficult to evaluate and to treat than other symptoms, such as pain 4. Indeed, it is often refractory to the point that the failure of systematic treatment requires emergency intervention, with the introduction of sedation in some cases 5,6. Several studies have reported frequencies for dyspnea, mostly for diverse stages of cancers 7–11 but also for noncancerous conditions 12–14. The heterogeneity of disease progression may partly account for the variability in the results obtained. Systematic reviews of the prevalence of symptoms have confirmed this variability at advanced stages of disease, for both cancers and noncancerous diseases 15,16. Combining the results from six studies, Teunissen reported a mean prevalence for dyspnea of 39% in the last 2 weeks of life, but with a very large 95% confidence interval (20–62) 16. Studies seeking an explanation for the origins of this variability, including, in particular, the effect of the underlying disease on the occurrence of dyspnea in the very last few days of life are rarer 17.

In our palliative care hospital (PCH), pain is regularly evaluated and tested, through daily determinations of pain scores based on self-reporting or evaluations by care staff. By contrast, there is no specific assessment of dyspnea, which remains difficult to measure and treat, despite a high level of awareness of the teams. We receive 1100 patients per year, mostly with terminal cancers and at the end of life. We therefore decided to investigate the occurrence of dyspnea at our center over the last 6 years. We measured its frequency, determined the timing of its occurrence in the last few days of life and tried to identify factors linked to the underlying disease associated with a predisposition to the occurrence of dyspnea at the extreme end of life. The care staff systematically noted all the patients’ symptoms in a prospective manner. This and the computerization of our files made it possible to carry out this large study.

## Methods

We analyzed all the medical records for patients hospitalized in the PCH of Maison Médicale Jeanne Garnier (France) during the last 6 years, without exception (no exclusion criteria). This observational study was approved by the appropriate regional ethics committee (*Comité de Protection des Personnes CPP Ile de France VIII*). The computerized prospective files contained administrative data and the care dossier, all medical observations, notes added by nurses, care assistants, and other paramedical staff, and all medical prescriptions. The data generated by the care management software (Osiris, from Corwin®, France) were extracted with a query tool (SAP® Business-Objects [BO] Web Intelligence, Levallois_–_Perret, France). For each stay in the PCH, we recorded the following data: age at admission, sex, dates of admission and discharge, and mode of discharge (transfer, death, return home), the site of the primary cancer and the presence or absence of lung metastases. We also collected data for patients with noncancerous diseases for the purposes of comparison. We searched the observations and notes obtained in a pilot phase for a preestablished stable list of terms: “dyspnea,” “out of breath/breathlessness,” “respiratory distress,” “breathing problems,” “suffocation/suffocating,” “asphyxia” and “finding it difficult to breathe/breathing badly,” excluding all negative forms (“no…” or “absence of…”); the frequency of each term used was recorded. These diverse terms (individual words or expressions) were then grouped together under the umbrella term “dyspnea.” The date of each observation was noted.

We identified patients who died after more than 6 days in the unit and analyzed them separately, to enable us distinguish acute dyspnea in the very last days of life from more precocious dyspnea. We classified all episodes of dyspnea on the basis of the time between the first episode noted during the patient's stay in the unit and death: exclusively terminal dyspnea (ETD) was defined as dyspnea first occurring in the last 4 days before the patient's death.

### Statistical analysis

The descriptive statistics are expressed as frequencies and percentages for categorical variables and as means and standard deviations (SD) for continuous variables. However, the duration of stays in the unit and the numbers of dyspnea episodes are expressed as medians and interquartile ranges (IQRs), due to the asymmetric nature of their distributions. We used chi-squared tests, Student's *t* tests, and Mann–Whitney *U*-tests for group comparisons, depending on the type of variable (qualitative, quantitative and ordinal, respectively). Contingency tables were used to estimate the crude relative risk or odds ratio (with a 95% confidence interval) for the occurrence of dyspnea. The threshold of significance was fixed at *P *=* *0.05 for two-tailed tests. We then carried out multivariate logistic regression analysis, with three explanatory variables for dyspnea (the site of the primary cancer, the presence of lung metastases and the site x lung metastasis interaction), to take into account the possible differential effects of metastases as a function of the site of origin. A second logistic regression analysis focused on the time of dyspnea onset, distinguishing between ETDs and more precocious episodes of dyspnea. Statistical analyses were carried out with XLSTat (version 5.01; Addinsoft, Paris, France) and R (http://www.r-project.org).

## Results

During the last 6 years, there were 6455 recorded stays in the PCH, corresponding to 6119 patients (92% with cancer): 242 patients had two stays in the PCH and 46 had three or more stays in the PCH. All the patients had a Karnovsky index of 40% or less and none of the cancer patients received chemotherapy or radiotherapy during their stay in the hospital. By performing queries (BO) on the 114,294 medical observations (0.8/patient per day) and 696,437 notes added by paramedical staff (4.8/patient per day), we identified 13,282 episodes of dyspnea occurring during 2608 stays in the hospital (5.1 episodes of dyspnea per stay). There was at least one episode of dyspnea during 40% of the stays in the hospital (Table1). Age, sex and having several stays in the PCH were not risk factors for the occurrence of dyspnea (Table1). The occurrence of dyspnea was linked to longer stays in the PCH: the median duration of PCH stay increased from 11 (4–24) to 17 (7–33) in patients experiencing dyspnea (*P *<* *0.0001).

**Table 1:** Demographic and pathological characteristics of the stays

	Total		Dyspnea		No dyspnea		Significance
	*N*	% in column	*N*	% in row	*N*	% in row	
Number of stays (%)	6455		2608	40	3847	60	
Age (±SD)	71.5 ± 13.7		71.2 ± 13.4		71.7 ± 13.8		*P *>* *0.05^1^
Men	2983	46	1213	41	1770	59	*P *>* *0.05^2^
Women	3472	54	1395	40	2077	60	
Single stay	5839	90	2376	41	3463	59	*P *>* *0.05^2^
Multiple stays	616	10	232	38	384	62	
Stay ending in death	5692	88	2341	41	3351	59	*P *=* *0.001^2^
Stay not ending in death	763	12	267	35	496	65	
Cancer	5932	92	2442	41	3490	59	*P *<* *0.001^2^
Disease other than cancer	523	8	166	32	357	68	
Median duration of stay (IQR): days	13 (5–27)		17 (7–33)		11 (4–24)		*P *<* *0.0001^3^

1 Student's *t* test.

2 Chi-squared test.

3 Mann–Whitney *U*-test.

The patient's file contained the term “dyspnea” in 53% of cases, “breathlessness” in 25% of cases, “respiratory distress” in 10%, “difficulty breathing” in 9%, and “asphyxia” in 2%. The doctors reported 28% of the episodes, the nurses and care assistants reported 66% of the episodes and physiotherapists and other paramedical staff reported 7% of the episodes.

### Influence of the underlying disease on the occurrence of dyspnea

Overall, 88% of all stays in the PCH ended in the death of the patient (Table1). Dyspnea occurred more frequently during stays ending in death (41%) than during other stays (35%; *P *=* *0.001). However, this difference corresponds to only a very small difference in the relative risk of death: 1.03 (1.01–1.05). Patients with cancers were at higher risk of dyspnea than other patients (RR of dyspnea: 1.30 [1.14–1.48]). Furthermore, mortality rates in the PCH were also higher for these cancer patients (RR of death: 1.35 [1.27–1.43]).

### Influence of the site of the primary cancer and of lung metastases

We determined the distribution of stays for cancer patients, as a function of the site of the primary tumor and the presence or absence of dyspnea (Table2). The four most frequent primary cancer sites accounted for 68% of the cancers: the lungs, digestive tract (10% colorectal and 17% other digestive sites), breasts, and urogenital area. The frequency of dyspnea depended on the site of the primary cancer, varying from 24% to 60%. Cancers of the esophagus, lung, breast, and ENT were associated with the highest frequencies of dyspnea. The cancers associated with the lowest risk of dyspnea were those of the brain, followed by digestive system cancers other than those of the esophagus, and urogenital cancers (e.g., cancers of the prostate and bladder). Lung metastases were recorded in 27% of stays. The cancers most frequently giving rise to these lung metastases were cancers of the skin, the breast and the colon/rectum. In patients with lung cancers, lesions at multiple sites were not always identified as lung metastases. In cases of lung metastasis, the risk of dyspnea was significantly higher, at 37–51% (RR: 1.37 [1.29–1.46]).

**Table 2:** Distribution of PCH stays for patients with a diagnosis of cancer, according to the site of the primary cancer and the presence or absence of lung metastases

					Cancer with lung metastasis				Cancer without lung metastasis			
	Stays		Stays with dyspnea		Stays		Stays with dyspnea		Stays		Stays with dyspnea	
Site of primary cancer	*N*	%	*N*	%	*N*	%	*N*	%	*N*	%	*N*	%
Esophagus	103	2	62	60	33	32	20	61	70	68	42	60
Lung/pleura	1093	18	625	57	239	22	148	62	854	78	477	56
ENT	307	5	148	48	80	26	37	46	227	74	111	49
Breast	734	12	336	46	321	44	193	60	413	56	143	35
Other/unknown/hematologic	457	8	193	42	118	26	67	57	339	74	126	37
Gynecologic	406	7	165	41	112	28	55	49	294	72	110	37
Skin	159	3	62	39	80	50	32	40	79	50	30	38
Urogenital	628	11	221	35	170	27	79	46	458	73	142	31
Other digestive cancer	1015	17	336	33	193	19	91	47	822	81	245	30
Colorectal	613	10	195	32	245	40	99	40	368	60	96	26
Brain	417	7	99	24	15	4	4	27	402	96	95	24
Total	5932	100	2442	41	1606	27	825	51	4326	73	1617	37

Cancers of the pleura or lung were grouped together. Cancers for which the primary site was unknown were grouped together with malignant hemopathies and cancers of “other” origins not classified elsewhere. PCH, palliative care hospital.

Multivariate logistic regression confirmed that the site of the primary cancer and the presence of lung metastases were factors significantly associated with the occurrence of dyspnea; the interaction was not significant. Table3 shows the odds ratios for the various primary cancer sites, taking brain cancers as the reference group. Colorectal sites were the only primary cancer sites with no more effect on the occurrence of dyspnea than brain cancer. By contrast, having lung cancer tripled the risk of dyspnea.

**Table 3:** Multivariate analysis: influence of the site of the primary cancer and of the presence of lung metastases on the occurrence of dyspnea, taking brain cancers as the reference group

	Odds ratio (95% CI)	*P*-value
Colorectal cancer	1.19 (0.89–1.59)	0.23
Other digestive cancer	1.44 (1.11–1.88)	0.006
Gynecologic cancer	1.9 (1.40–2.58)	<0.001
ENT cancer	2.62 (1.90–3.62)	<0.001
Lung cancer	3.88 (3.00–5.02)	<0.001
Breast cancer	2.14 (1.62–2.81)	<0.001
Urologic cancer	1.51 (1.14–2.00)	0.004
Lung metastasis	1.81 (1.59–2.07)	<0.001

### Differential influence of the site of the primary cancer on ETD and more precocious dyspnea

**Figure 1:**
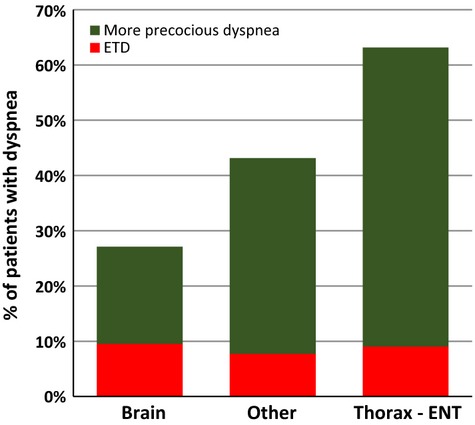
Extracted data for 3621 cancer patients who died after more than 6 days in the palliative care hospital. Dyspnea occurred in 47% of these patients. Cancers were classified into three groups according to the frequency of dyspnea: low frequency (brain: 27%), high frequency (thorax and ENT: 63%), and intermediate frequency (others: 43%). Dyspnea was classified according the timing of the first episode: during the last 4 days of life (exclusively terminal dyspnea: ETD) or earlier (more precocious dyspnea). The frequency of ETD (8–10%) was independent of the site of the primary cancer, unlike that of more precocious dyspnea (18–54%).

We carried out an analysis of the 3621 cancer patients who died after more than 6 days in the unit: 47% had dyspnea, 8% classified as ETD (dyspnea first occurring in the last 4 days before death), and 39% occurring earlier with respect to the patient's death. As illustrated in Figure1, the various types of cancers were classified into three groups, according to the frequency of dyspnea: rare (brain cancer), highly frequent (lung/pleura, esophagus and ENT cancers), and moderately frequent (all other cancers). The frequency of the more precocious form of dyspnea clearly differed between primary cancer sites (varying from 18% to 54%) whereas the frequency of ETD was almost constant for the various sites (8–10%); this difference was statistically significant (*P *<* *0.001).

## Discussion

This study confirms the high incidence of dyspnea (41%) in a population of cancer patients in a PCH with a mortality rate of 88%. However, this incidence depends strongly on the initial disease: it varied from 24% to 60% for patients with cancers, depending on the primary site of the cancer. By contrast, 8% of the patients first experienced dyspnea during the last 4 days of life. The frequency of such ETD was not dependent on the underlying disease.

### Comparison of the frequency of dyspnea with the values reported in other studies

Our results are consistent with the variability in values reported in previous studies, ranging from 21% to 70%. This variability may reflect the mode of data recording (by self-reporting or observation) and the cutoff points used to define the intensity of dyspnea. Dudgeon reported a frequency of 46% for a population of 923 patients with cancer who were not hospitalized, and identified certain risk factors, such as smoking, asthma, chronic obstructive bronchitis, and pulmonary radiotherapy 9. In a prospective study of 135 patients with advanced cancers, Bruera et al. reported scores for dyspnea of at least 30 on a self-report scale running from 0 to 100 in 55% of the patients 8.

### Increase in the frequency of dyspnea at the end of life

We found that dyspnea was more frequent during hospital stays ending in the patient's death than during stays with other outcomes. Several studies have reported an increase in the frequency of this symptom during the last few months of life 7,17–19. This is consistent with findings suggesting that the occurrence of dyspnea is a factor predictive of death 20,21. Nevertheless, in our study, the occurrence of dyspnea was significantly associated with death, but had little clinical impact, because the relative risk was only 1.03. It does not therefore appear to be possible to establish a systematic causal link between dyspnea and death. Even though dyspnea had a little influence on the relative risk of death, it is important to take it into account considering the patients’ anxiety and suffering. Indeed, we should instead distinguish between two populations of dyspneic patients: those in whom dyspnea is a subacute or chronic phenomenon and those in whom the first episode of dyspnea occurs during the four last days of life (ETD). Our data suggest that the frequency of ETD (8%), unlike that of more precocious forms of dyspnea, is independent of the underlying disease. This distinction may be of particular importance for therapeutic management. During the last few days of life, treatment is exclusively symptomatic, with particular attention paid to death rattles when patients are too weak to expectorate and have reduced levels of consciousness. Before this period, patients with subacute or chronic dyspnea could potentially benefit from treatments more closely adapted to their physiopathology. Some such patients with particular indications might also benefit from noninvasive ventilation, as recently suggested, but the use of this technique immediately raises the broader question of the quality of life of patients ventilated in this way 22. Conversely, for patients with dyspnea in the final phase of their disease, oxygen treatment, which is frequently administered in practice, might actually be ineffective and may even be deleterious in terms of patient comfort 23. Thus, clinical differentiation between these two categories of patients, with the accurate identification of those in the final phase, could lead to the development of more appropriate therapeutic strategies.

### Prolongation of the hospitalization of patients with dyspnea and dyspnea duration

The median duration of stay in the PCH was longer for patients experiencing at least one episode of dyspnea during their stay. This result may be explained, in part, by the patients presenting “chronic” dyspnea before the last week of life, potentially leading to admission to the PCH or hindering the maintenance of the patient at home or their return home, due to the risk of asphyxial respiratory distress. For other patients displaying dyspnea exclusively at the end of their stay, the longer stay in the PCH may reflect a prolonged state of fragility, with cachexia, major asthenia, and confinement to bed for a long period. Cachexia, muscle wasting, and fatigue are factors likely to play a key role in the occurrence of episodes of dyspnea at the end of life 24. Other authors have stressed the importance of the multidimensional nature of “dyspnea” as a symptom, reducing the importance of the role of organic factors, to take into account more broadly psychological (anxiety, depression) social, and even spiritual factors 10,25.

Cancer has already been identified as a risk factor for dyspnea. The site of the primary cancer played a key role in the risk of dyspnea before the last 4 days of life, confirming not only the risk associated with pleural/pulmonary cancer already reported in other studies, but also a risk for other thoracic cancers (esophagus, breast) and ENT cancers 8,19,26. The data available for PCH patients are not detailed enough for analysis of the correlation between lesion extent and dyspnea. It is difficult to characterize pulmonary and pleural lesions in a PCH, where the principal objective is controlling symptoms, rather than carrying out an extensive radiological evaluation of the disease. By contrast, some cancers, such as brain cancers and colorectal cancers, only rarely lead to dyspnea. According to our data, 27% of the cancer patients hospitalized in the PCH had lung metastases. The type of cancer most frequently displaying metastasis to the lungs at the end of life was melanoma (50%), followed by breast cancer (44%). Our results indicate that not all lung metastases are equivalent in terms of their clinical impact. In this respect, it would probably be interesting to identify specifically those patients suffering from carcinomatous lymphangitis, as we have observed that this disease frequently causes severe dyspnea.

### Limitations of this study

In this retrospective study, dyspnea was reported only in observations or medical notes, and only in terms of its occurrence or absence, without details of self-reported or observation scales and scores. It was not, therefore, possible to assess the intensity of dyspnea in these patients, the duration of each episode nor the successful of the treatments. Indeed, within our institution, pain is the only symptom for which traceability has been established through a daily evaluation system (self-reporting if possible, observation otherwise). Nevertheless, the recording of the occurrence of dyspnea in medical records or nursing notes indicates that this symptom was sufficiently severe to have been noticed and to have required management. Thus, the reported events were clearly clinically “significant” events rather than low-intensity events not necessitating a modification of treatment or patient management. Our data therefore probably tend to underestimate the frequency of this phenomenon, because less severe episodes probably went unrecorded. This underestimation may have been further accentuated by the widespread use of opioids and anxiolytics in the PCH, a combination that has been shown to be effective at reducing the sensation of dyspnea 1,2,27–30. It is particularly interesting that most of the dyspnea episodes occurred in the last few days of life, a period during which 70% of patients have opioid treatment, which may mask some dyspnea episodes (F. Guirimand; pers. data; published as an abstract SFAP 2011). A correlation has also been found between dyspnea and pain in patients with advanced cancers 10. However, a prospective study, with periodic, systematic, standardized assessments, is required to provide more definitive answers to these questions.

Other authors have reported that there is a risk of underestimating dyspnea 31; it has been found, for example, that 23% of cancer patients with a fortuitously discovered pulmonary embolism actually experience dyspnea without this symptom being noticed by doctors 32. Our results thus suggest that it would be beneficial to implement a systematic scale for assessing dyspnea, beginning at admission, with systematic re-evaluations during the patient's stay. However, self-report scales, such as the Edmonton or the French-validated MDASI scales, may not always be appropriate for use in patients in the terminal phase of illness, because 70% of these patients experience cognitive problems or a loss of vigilance in the last few days of life 7,27,33,34. Observation scales for dyspnea are therefore of particular importance for use in this population and their validation in French is urgently required 35–37.

Another limiting factor is the lack of exhaustivity of data concerning preexisting pulmonary factors (chronic obstructive pulmonary disease, asthma, smoking), which undoubtedly affect the risk of dyspnea at the end of life. Similarly, clinical examination data (auscultation, presence or absence of pleural discharge) were not available for this study. Finally, the presence or absence of metastases was determined on the basis of radiological examinations carried out, in some cases, several weeks before admission to the PCH. This lack of recent examination data may have biased the data analysis, because it was not possible to update the available data in the PCH.

In conclusion, the first step toward the effective management of dyspnea in a PCH is the recognition of its very high prevalence: this condition affects at least 24% of cancer patients in the absence of a predisposing conditions (brain cancer), and as many as three in five patients with thoracic cancers. For 8–10% of patients, dyspnea occurs only in the last 4 days of life, regardless of the site of the primary cancer. At this time, changes in vigilance occur, requiring the implementation of systematic evaluation based on the use of an appropriate observation scale. Future clinical studies should distinguish between episodes of dyspnea occurring in the very last days of life and more precocious episodes of dyspnea, because these two types of dyspnea may require different management practices.

## Conflict of Interest

None declared.
